# The way we look at an image or a webpage can reveal personality traits

**DOI:** 10.1038/s41598-024-62210-z

**Published:** 2024-07-05

**Authors:** Thomas Le Bras, Benoit Allibe, Karine Doré-Mazars

**Affiliations:** 1 Vision Action Cognition Laboratory, Université Paris Cité, 92700 Boulogne-Billancourt, France; 2 Research and Development Department, AB Tasty, 75013 Paris, France

**Keywords:** Human behaviour, Personality, Saccades

## Abstract

Personality is a central concept and a cross-domain explanatory factor in psychology to characterize and differentiate individuals. Surprisingly, among the many studies on oculomotor behavior, only a few have investigated how personality influences the exploration of a visual stimulus. Due to the limited number of existing studies, it is still uncertain if markers of personality in eye movements are always observable in eye movements across various exploration contexts. Here, introducing a novel concept of gaze-based signatures of personality, we used visual exploration metrics to detect personality signatures across various exploration contexts (visual search and free-viewing on images and webpages) in 91 participants. Personality data were collected as in the reference paper that validated the French version of the Big Five Inventory. Linear regression analyses demonstrated that while Extraversion and Openness to Experience did not correlate with any particular exploration metric, the other three traits–Conscientiousness, Agreeableness, and Neuroticism–correlated robustly with all exploration metrics in different visual exploration contexts. Our study provides evidence for the capture of the gaze-based signature of personality from very brief eye movement recordings.

## Introduction

Visual exploration enables us to gather crucial information that helps us understand and adapt to our surroundings. While appearing to be a natural and straightforward behavior, visual exploration is intrinsically complex, influenced by a myriad of factors. To fully understand visual exploration, we need a comprehensive description of its characteristics and nature. Additionally, understanding the extraction of visual information requires delving into various processes, from physiological to cognitive or neuro-cognitive. All of these processes are far from having been described and explained, and we know little about how these processes interact with each other. What we do know is that exploring a visual scene consists of a series of alternations between rapid eye movements, known as saccades, and moments of arrested stare called fixations. Although we pay scant attention to it, it is through more than 170,000 alternations between saccades and fixations that we extract visual information in our daily lives^1^. Saccades, which direct the fovea to interesting parts of the scene, are considered to be driven by visuo-spatial attention. The location where eyes land at the end of the movement depends on where attention (whether endogenous or exogenous) has been directed. Fixations, on the other hand, are directly related to information processing, as they allow us to position the fovea on a local area of the visual stimulus to extract all the essential information. Also, with the aim of optimally processing visual information, visual exploration is dynamic and changes over time based on the type of information we process. Specifically, during visual exploration we alternate between two modes of processing:ambient and focal modes. The ambient mode allows us to obtain the gist and spatial organization of the scene and after the first few seconds of visual exploration in ambient mode, we switch to the focal mode which allows us to identify and process visual information in depth^2–5^. The ambient mode is primarily driven by the dorsal pathway, associated with exogenous attention, characterized by shorter fixation durations (measured in milliseconds) and larger saccade amplitudes (measured in degree of visual field)^2^. Information gathered by the ambient mode is processed in a largely unconscious manner. In contrast, the focal mode is primarily driven by the ventral pathway and is linked to endogenous attention, with longer fixation durations and smaller saccade amplitudes, and the information collected in this mode is processed consciously^2^. Several studies have indicated that there is almost always this initial mode change from ambient to focal mode at least in the case of image viewing experiments, but some recent studies have also revealed that during visual exploration, several mode shifts occur at different moments, showing that visual exploration is not stable and identical over time^5^.

In general, visual exploration is influenced by two types of factors:bottom-up factors, such as the overall shape of the stimulus, and top-down factors, such as the goal of the visual exploration. Many bottom-up factors have been highlighted, especially in numerous computational modeling studies of visuo-spatial attention^6,7^. Although it is complex to distinguish the various bottom-up characteristics, studies have already revealed the existence of these elements and even used them to create visual saliency maps^6–8^. Relying solely on this low-level stimulus information, numerous research efforts have shown the possibility of partially predicting eye movement by considering information such as color contrasts, orientations and intensity^6,8^. However, among the studies focusing on these bottom-up factors, few have looked at the new types of stimuli flooding our daily lives:websites. We know, for instance, that due in part to the mix of specific elements that make them up, such as images and text, websites have a global architecture that is very different from the images typically used in eye-tracking studies^9^. A few studies have already shown that exploring a website is more complex than exploring an image^10^. These stimuli remain less well studied than images, even though their nature is very different from that of the images used to gain insight into the complexity of visual exploration. Regarding top-down factors^11^, the objective of visual search remains one of the most widely studied factors in the literature^12^. This factor appears to be one of the most robust across studies and proposed tasks. It has been shown that certain metrics of oculomotor behavior are related to the task. The most closely related metrics seem to be temporal such as fixation durations and spatial such as saccade amplitudes^12^. Specifically, certain studies have shown that eye movement metrics can be categorized into three distinct parts:information related to fixation positions, which are spatial data, the duration of the fixations made by individuals, which are temporal data, and their dynamics, i.e. how these two types of information spatial and temporal - evolve over time^12–14^. Generally, it appears that as the complexity of a task increases, fixation durations also increase. In fact, visual information processing times seem to be correlated with the level of interest, depth, and complexity of task execution or stimulus processing^15,16^. We know, for instance, that other factors related to individual characteristics such as expertise about the content of the visual stimuli^17^, the age of the person processing the visual information^18^, or personality^19^, can influence visual exploration.

Personality is one of the most well-known concepts in the psychology literature, yet it has seldom been studied in eyetracking. One of the most consensual models of personality is the Big Five Factors Model^20^. It defines personality based on five traits:Openness to Experience (labeled O), Conscientiousness (C), Extraversion (E), Agreeableness (A), and Neuroticism (N)^20^. Together, these personality traits account for the majority of the characteristics that can describe and categorize individuals. Based on these 5 dimensions, an individual’s personality can be summed up by placing the person on a 5-point Likert scale for each personality trait. Individuals with a high score in Openness to Experience are more inclined to seek out new experiences and be creative rather than sticking to their usual routines. People who score high in Conscientiousness are more organized and responsible, rather than neglectful and disorganized. Those high in Extraversion lean towards being sociable and energetic, as opposed to reserved and introverted. Individuals with high scores in Agreeableness are compassionate and cooperative, instead of confrontational and antagonistic. Lastly, those with high scores in Neuroticism tend to experience negative emotions and anxiety, in contrast to being emotionally stable^20^. Personality has been frequently associated with both general behaviors such as job^21^ or academic performance as shown in meta-analyses combining 15 studies and cumulative samples exceeding 70,000 individuals^22^. Even words chosen in a music writing task^23^ or in cognitive abilities^24^ have been linked with personality. In the latter study, Sutin et al.^24^, asked participants to complete the five-factor model personality test and a cognitive battery that measured performance in five domains:memory, speed-attention-executive, visuo-spatial ability, fluency, and numeric reasoning. They showed that a high level of Neuroticism was linked to poorer performance across all cognitive tasks. Conversely, higher Openness to Experience and Conscientiousness typically correlated positively with better performance, though not uniformly across all tasks. Extraversion showed a positive association only with the speed-attention-executive and fluency domains, and no relation to others such as episodic memory. Surprisingly, Agreeableness was connected to better performance in all domains except numeric reasoning. More specifically, another study^25,26^ demonstrated that the traits of Conscientiousness and Openness to Experience were particularly involved in attentional processes. Participants who scored higher on Conscientiousness demonstrated superior performance on attentional tasks compared to less conscientious individuals. On the other hand, it has been shown that individuals who scored higher on Openness to Experience displayed enhanced mental flexibility^26^. In addition to these links with attentional processes, some studies have shown links between personality and eye movements^19,27–32^.

For instance,^27^ demonstrated that links between the traits of Agreeableness and Conscientiousness had an influence on information processing time and the dispersion of visuo-spatial attention. In their study, they clustered participants based on their personality traits into two groups: one group with a dominant trait of Agreeableness and another with a dominant trait of Conscientiousness. Their results showed that for almost all their conditions testing preferences for bottom-up information such as color, text layout, or alignment of elements composing a visual stimulus, the fixation duration average was longer for the Agreeableness group compared to the Conscientiousness group. The authors interpreted this to mean that individuals with a higher score of Conscientiousness processed visual information faster through their eye movements compared to those with a high score of Agreeableness. In another study,^33^ showed that it was possible to establish a classification algorithm to predict individuals’ personality based solely on their eye movements while exploring images and videos. The results showed that prediction performance was higher for video stimuli, which are dynamic, compared to images which are static. This provided additional evidence that interactions exist between top-down factors, such as personality, and bottom-up factors. However, some studies seem to show that the context, and specifically the task given to a participant, influences the ability to express personality. A study examining the relationship between eye movements and personality traits^34^ asked participants to carry out three types of visual search tasks: factual, exploratory, and interpretative. It was found that in the context of a factual task, eye movements correlated most strongly with the personality traits of Conscientiousness, followed by Agreeableness, and then Extraversion. However, in an exploratory task context, this hierarchy shifted. In this setting, Extraversion, Agreeableness, and then Conscientiousness emerged as the traits exerting the most significant influence on eye movements. The results also showed that the expression of the Extraversion, Agreeableness, and Conscientiousness traits through eye movements depends on the context in which the visual exploration occurs. Furthermore, a significant portion of the literature has employed machine learning techniques^19,28,29,33,35^. Although these methods have successfully predicted personality from eye movements, clearly indicating a link between personality and eye movements, there still exists some ambiguity regarding the nature of these effects. For instance, it is not clear whether an increase in a personality trait score (Conscientiousness for example) leads to an increase or decrease in values of eye movement indicators (such as fixation durations for example). Likewise, the specific relationships between eye movement metrics and personality traits are yet to be precisely defined. We do not know whether indicators related to the depth of visual information processing (such as fixation durations) are more strongly associated with personality scores than indicators of the scope of visuo-spatial attention deployment (such as saccade amplitudes).

In the realm of neuroscience, studies have demonstrated the existence of neuroanatomical and functional differences among individuals with varying scores. Specifically, drawing upon the framework of the “Biological Theory of the Big Five Personality Traits”^36^ various associations between the big five personality traits and brain structures have been demonstrated^36^. Notably, Openness to Experience has been found to exhibit links within a region of the parietal cortex engaged in working memory and attention control. Conscientiousness has been positively associated with the volume of the middle frontal gyrus in the left lateral prefrontal cortex, a critical region for both working memory and attention. Extraversion has shown an association with the volume of the medial orbitofrontal cortex, which is particularly involved in decision making and attention to emotional stimuli^36^. Changes in volume in the left posterior superior temporal sulcus and the posterior cingulate cortex, regions intricately engage in processing social information, have marked Agreeableness. Finally, Neuroticism has been linked to volume differences in the hippocampus, middle cingulate cortex, and dorsomedial prefrontal cortex, indicative of heightened sensitivity to threats related to emotion dysregulation. Commonalities can be found between brain regions involved in personality and those engaged in eye movements and visuospatial attention. Indeed, frontal eye fields, supplementary eye fields, and the posterior parietal cortex are activated during voluntary shifts of attention. These regions, along with the dorsolateral prefrontal cortex, play a role in voluntary saccades and spatial working memory. The cortical control of saccade sequences, including simple saccades and their coordination with the precuneus in the intraparietal sulcus, as well as regions anterior to the supplementary eye fields and anterior to the frontal eye fields in the superior frontal sulcus, has been explored^36^. Also, the basal ganglia are particularly involved both in the expression of personality^37,38^ and in oculomotor behavior^39,40^. Specifically, research showed that the basal ganglia^37^ play a significant role in the control of voluntary motor movements, emotion, and cognition. The involvement of this structure is hypothesized to occur through its multiple connections. The basal ganglia are interconnected with the thalamus, the motor cortex, and the prefrontal cortex. The prefrontal cortex has been shown to be responsible for personality expressions and the planning of complex cognitive behaviors^41^. The premotor cortex, which is a component of the motor cortex, is strongly associated with continuous motor movements, and the dynamic activities in neuronal populations of the premotor cortex are stimulated by decision-making processes^42^.

Together, all these results suggest the possibility of observing a new concept that we introduce here: Gaze-based Signatures of Personality (GSP). Since personality is a significant high-level factor, if GSPs exist, we should be able to observe them through oculomotor behavior metrics that are sensitive to high-level factors. However, it remains unclear whether GSPs can be robustly observed across different visual exploration contexts such as on web pages and constantly throughout the visual exploration, regardless of when in the exploration we observe the signature. Therefore, the goal of the present study was to detect GSPs over time by manipulating top-down (tasks) and bottom-up (stimuli types) factors. Like any signature from a signal, it is essential to assess its robustness, i.e. how detectable it remains across various contexts and among different noise levels, and its constancy, i.e. how identical the signature remains across different measurements. Also, given the complexity of personality and its multi-domain influence on behavior, it is crucial to diversify the information measuring its signature in order to boost the signal-to-noise ratio and accuracy. Visual exploration is dynamic and the information processed during visual exploration also evolves over time. As we progress through visual exploration, we gain knowledge about the visual scene, which in turn influences subsequent visual exploration behavior. It is essential to consider how top-down factors change over time^12^ and given that personality is a top-down factor, it is evident that its influence on visual exploration also varies over time. From this perspective, it is possible that GSPs may be time-dependent. To our knowledge, this aspect remains underexplored in current research. Beyond the interplay between top-down factors such as task and personality, it is crucial to understand how bottom-up factors influence the gaze-based signature of personality. Hence, results for images and web pages will be analyzed separately due to their distinct nature. Specifically, our ambitious objective is to detect if visual exploration patterns strongly correlate with changes in personality scores for a given trait. We conducted analyses trait by trait, attempting to identify significant differences related to personality scores in eye movements. To achieve this, we employed various visual exploration metrics, some related to cognitive processing and others to spatial information, which we present in more detail in the results section. Given the exploratory nature of this field, we did not formulate precise a priori hypotheses except that, since Conscientiousness, Agreeableness, and Extraversion are the traits with the most effects on eye movements, we expect to observe the most GSPs on these personality traits^34^. Instead, with the aim of framing and identifying potential personality signatures, we established a prerequisite. The prerequisite is that we anticipate observing different behaviors depending on the task, stimulus, and over time. This initial prerequisite will ensure that we have placed participants in varied visual exploration contexts and that any detected GSP occurs in diverse settings. By definition, personality is assumed to be constant across time and domains. Therefore, to claim a personality signature, the associations we observe between personality traits and eye movements have to persist across different contexts.

For all these reasons, we propose the following definition of GSP: An Gaze-based Signature of Personality (GSP) is a set of common oculomotor patterns (i.e., markers) associated with personality traits constant and is robust across various visual exploration contexts. These GSPs enable the detection and comprehension of how personality influences eye movements, including their direction and intensity, affecting both visual information processing and the deployment of visuo-spatial attention.

## Results

### The role of context effects: task, stimulus type and time effects

First, to assess how our factors induce different visual exploration behaviors and to verify the existence of varied exploration contexts, we evaluated the influence of contextual factors on eye movement metrics such as the task (free-viewing vs. target-finding), the type of stimulus (images vs. web pages), and the time (bins of one second). In this section, we will briefly present the metrics used and the observed effects. Our categorization of metrics was inspired by^12^. For all the subsequent analyses, both simple and multiple linear regressions were used. The interpretation is based on the *β* regression coefficient, which indicates the direction and strength of the effect. For instance, if we test the effect of time (as independent variable) on fixation duration (as a dependent variable), a significant regression coefficient equal to 42 means that an increase of one unit (one second) is associated with an increase of 42 ms in the fixation durations. A significant regression coefficient is equal to 42 means that an increase of one unit (one second) in the independent variable is associated with a decrease of 42 ms in the fixation duration. Moreover, for categorical independent variables, such as task type or stimulus type, an increase of 1 on the independent variable scale should be understood as moving from the free-viewing task to the target-finding task, and transitioning from images to web pages respectively.

#### Spatial information

**Figure 1:**
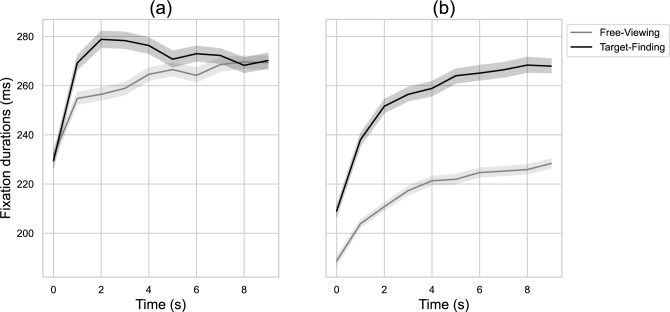
Mean fixation durations over time on images (**a**) and web pages (**b**) with a 95% confidence interval.

The *dispersion of fixations* is a metric calculated from the standard deviation of the x and y fixations positions, that measures the spatial overlap of the processed information. The greater the dispersion of fixations the more extensive the space over which the visual exploration was carried out. *Saccade amplitudes*, measured in degree of visual field, are among the most frequently used metrics in the literature. They measure the dispersion of visuo-spatial attention and provide information on the average distance over which attention was directed before making a fixation. We found a significant effect of the task on the horizontal dispersion of fixations with *β* = − 17.57, *p* < 0.001. This indicates that the dispersion of fixations on the horizontal axis was 17.57 pixels less in the target-finding conditions compared to the free-viewing conditions. Tasks also had a significant impact on the vertical dispersion of fixations with *β* = − 20.4, *p* < 0.001. The type of stimulus showed a significant influence on the horizontal dispersion of fixation, *β* = − 130.22, *p* < 0.001 and on the vertical dispersion of fixations, *β* = 44.14, *p* < 0.001. The latter indicates that the dispersion of fixations on the vertical axis was 130.22 pixels less in the web page conditions compared to the image conditions. Finally, we also observed a significant time effect on the vertical dispersion of fixations, *β* = 5.5, *p* < 0.01. The task had a significant effect on saccade amplitudes, *β* = 0.07, *p* < 0.001. Likewise, the type of stimulus significantly influenced saccade amplitudes, *β* = − 0.16, *p* < 0.001. Finally, a significant effect of time on saccade amplitudes *β* = − 0.07, *p* < 0.001 was found, indicating that, with each passing second during visual exploration, saccade amplitudes decreased by 0.07° of visual field, as shown in Fig. 1.

#### Information processing

*Fixation durations*, are also among the most commonly used metrics in the literature. They are closely related to the depth of processing carried out and the interest in an element or the difficulty of a task. The task significantly influenced fixation durations, *β* = 23.6, *p* < 0.001. The type of stimulus also had a significant effect, *β* = − 29.82, *p* < 0.001, as did the time *β* = 2.87, *p* < 0.001 (see Fig. 1).

#### Dynamics of the ambient/focal modes of information processing

The time spent in ambient mode is a metric derived from the K coefficient^5^. This metric is measured in milliseconds and provides insight into the duration dedicated to spatial and superficial processing in relation to the exploration duration analyzed.

The number of mode switches, also derived from the K coefficient, is a metric informing about the stability of visual exploration behavior. Results showed that the task significantly influenced the time spent in ambient mode, *β* = − 249.03, *p* < 0.001 and the number of mode switches, *β* = − 3.09, *p* < 0.001. The type of stimulus showed a significant effect on the number of mode switches, *β* = 2.24, *p* < 0.001. We observed a time effect on the K coefficient, *β* = 0.04, *p* < 0.001, and the number of mode switches, *β* = 2.12, *p* < 0.001.

#### Conclusions

These results show that visual exploration in target-finding conditions is more related to deep processing, dispersed visuo-spatial attention, shorter ambient mode duration, and thus a tendency to spend more time processing information deeply while making less dispersed fixations. These initial analyses show that the effect of the task is substantial since we observed an effect on almost all the eye movement metrics. Analyses concerning the stimulus effect showed that there was a significant effect on the time spent in ambient mode, the number of mode switches, and the dispersion along the Y-axis, with higher values on web pages than on images. Conversely, fixation durations, saccade amplitudes, and dispersion along the horizontal axis were significantly lower on web pages than on images. Web page visualizations tend to spend more time in ambient mode, be less stable, and be more dispersed, covering more surface along the Y-axis. Analyses also showed that visual explorations were associated with shallower processing, with less dispersed visuo-spatial attention and less extensive processing on web pages. Like the task type, the stimulus type significantly influenced visual exploration behavior. Given that we found a significant effect of task, stimulus, and time on almost all metrics, this means that they are key influencing factors to consider since they induce varied and distinct visual exploration behaviors. In light of these findings, the following analyses were conducted by considering these different contexts of visual exploration separately and accounting for its dynamic evolution over time.

### Scores of personality traits

**Figure 2:**
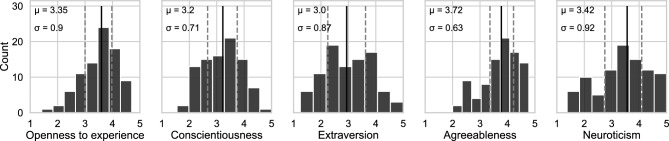
Distribution of personality scores.

We then looked at the personality trait scores obtained from the questionnaires. First, we ensured that the distribution of scores for each trait was sufficiently dispersed to represent a diverse data set. Additionally, we wanted to ensure that the data obtained were comparable to those of a reference study^43^, for the French validation of the Big Five Inventory 45. The statistical distribution for each of the traits of 91 participants can be seen in Fig. 2. The score distribution is varied enough to be used in subsequent analyses, and also closely matches the reference paper, which was based on over 2499 individuals^43^:the values obtained in this reference paper for the trait Openness to Experience (3.5 ± 0.6), Conscientiousness (3.4 ± 0.7), Extraversion (3.2 + 0.8), Agreeableness (3.9 ± 0.6), and Neuroticism (3.0 ± 0.8) are quite similar to ours, as shown in Fig. 2. As previously explained, we used multiple linear regressions to study the effects of personality on eye movement metrics in order to remain as close as possible to the initial theoretical framework of the Big Five model, which uses continuous scales to characterize an individual’s personality within the model’s five dimensions.

### Interaction effects between personality traits and social and emotional contents

In order to ensure that the observed effects of personality traits are not influenced by secondary factors such as emotional and social content, we conducted additional analyses. These analyses assessed the interaction effects of personality traits with valence and social agents on eye movement indicators. Similarly, the interpretation is based on the *β* regression coefficient. An increase of 1 on the categorical independent variable scale should be understood as a difference of the effect of the personality trait tested in the interaction moving from no social to social content or no emotional to emotional content. The analyses were conducted on the entire trial. All significant interaction effects observed were only on images in the target-finding task. We observed an interaction effect between valence and the Conscientiousness trait on time spent in ambient mode, *β* = 224.15, *p* < 0.05 and the number of mode switches, *β* = 0.98, *p* < 0.05. Another interaction effect was observed between valence and trait Agreeableness on saccade amplitudes, *β* = 0.19, *p* < 0.05. Interaction effects were also observed between social content and trait Conscientiousness on the number of mode switches, *β* = 1.07, *p* < 0.05, and trait Agreeableness on fixation durations, *β* = 8.02, *p* < .05). The analyses of simple effects showed that there was an effect of Conscientiousness on the number of mode switches in the absence of social content (B = − 1.11, *p* < 0.05 and in the absence of emotional content, *β* = − 1.01, *p* < 0.05. Further analysis of simple effects from interaction effects showed a significant effect of the Conscientiousness trait only on the number of mode switches. Specifically, simple effects were observed with stimuli with no social content, *β* = − 1.1, *p* < 0.05 or no emotional content, *β* = − 1.02, *p* < 0.05. Further analysis of simple effects from interaction effects showed a significant effect of the Conscientiousness trait only on the number of mode switches. Specifically, simple effects were observed with stimuli with no social content, *β* = − 1.1, *p* < 0.05 or no emotional content, *β* = − 1.02, *p* < 0.05 .

### Signatures of personality over time and robustness

**Figure 3:**
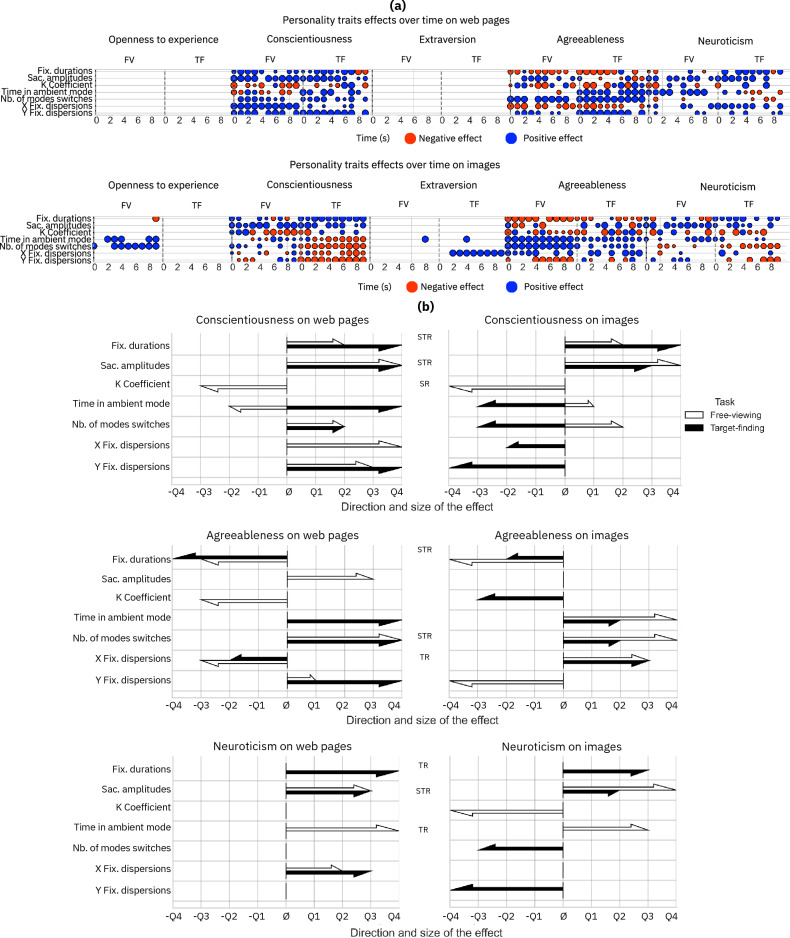
Effects of personality traits on eye movement indicators for each of the two tasks and on both types of stimuli. The upper section (**a**) highlights the magnitude and direction of the significant (*p* < 0.005 after Bonferroni correction) personality trait effects for each metric and personality trait, symbolized by the size (magnitude) and color (direction) of the dots. Specific timestamps in seconds are provided at the bottom of each section. FV and TF correspond respectively to Free-viewing and Target-finding conditions. The lower section (**b**) summarizes the significance of personality traits on each metric (*TR* task robustness, *SR* stimulus robustness, *TSR* task and stimulus robustness). If the effect of a personality trait is consistent across both tasks, it is considered robust to the task. If it is consistent (i.e., with the same direction) across both stimuli, it is considered robust to stimuli, and if it is consistent across both, it is considered robust to both stimulus and task. The size of the effects is represented by the length of the arrows.

Given that the analyses based on chunking the data by trial did not reveal sufficient GSPs for each experimental condition, we focused on analyzing the data by taking into account the dynamic evolution of visual exploration. To achieve this, we recalculated all the metrics over time by choosing a chunking time window of one second. The goal was to study visual exploration metrics in a given task, stimulus and moment. As these analyses correspond to multiple comparison analyses, Bonferroni corrections^44^ were performed on the *p*-values. The results are shown in Fig. 3. Effect size values are also given in Table 1. We first present the two traits showing the least GSPs, namely Extraversion and Openness to Experience, and then the other three traits, Conscientiousness, Agreeableness and Neuroticism, which exhibited clear GSPs across visual exploration contexts and time. After conducting a detailed analysis for each stimulus and task observing effects at each second, the results were synthesized in Fig. 3 (part b). The examination focused on how each personality trait impacted eye movements during the 10-s study period. When significant effects persisted for the majority of the time (i.e., 5 s or more) and remained consistently in the same direction, it was concluded that the effect of the given trait on the specific eye movement indicator could be considered as positive if it was present and positive for at least 5 s out of 10, and negative if it was present and negative for at least 5 s out of 10. Subsequently, an assessment was made to determine whether the effects of personality traits on eye movement indicators exhibited persistence and consistency in direction across both tasks (task robustness), both stimuli (stimulus robustness), or both tasks and stimuli (task and stimulus robustness). The results of the analyses conducted across the 4 conditions (free-viewing on images, target-finding on images, free-viewing on web pages and, target-finding on web pages), will be detailed. Findings from images will be discussed initially, followed by those from web pages. Traits showing the fewest effects (Openness to Experience and Extraversion) will be presented before the three other traits showing GSP on some eye movement indicators. Overall, the obtained results showed that the traits Openness to Experience and Extraversion do not contain GSPs while the traits Conscientiousness, Agreeableness and Neuroticism do.

**Table 1:** *β* values for free-viewing task on images (a), target-finding task on images (b), free-viewing task on web pages (c) and target-finding task on web pages (d).

	(a)						(b)					
	Q1	Q2	Q3	Q4	Mean	Std	Q1	Q2	Q3	Q4	Mean	Std
Fixation durations (ms)	1.50	4.93	8.57	14.40	7.73	5.80	0.84	3.89	6.54	10.02	5.63	3.89
Saccade amplitudes (° vf)	0.01	0.06	0.10	0.18	0.12	0.05	0.02	0.06	0.10	0.15	0.09	0.05
Coefficient K	0.01	0.02	0.03	0.07	0.04	0.04	0.00	0.01	0.03	0.05	0.03	0.02
Time spent in ambient mode (ms)	8.27	52.62	98.12	139.18	77.22	58.13	13.76	27.50	43.50	73.49	39.22	24.64
Number of modes switches	0.16	0.44	0.93	1.80	0.85	0.76	0.10	0.31	0.43	0.62	0.46	0.16
Fixation dispersions on X axis (pixels)	0.28	1.18	3.03	5.48	2.51	2.20	1.27	3.58	5.50	11.48	5.78	4.80
Fixation dispersions on Y axis (pixels)	0.37	1.23	2.22	3.94	1.95	1.57	0.39	1.38	2.48	3.69	2.08	1.35

	(c)						(d)					
	Q1	Q2	Q3	Q4	Mean	Std	Q1	Q2	Q3	Q4	Mean	Std
Fixation durations (ms)	0.55	1.32	2.56	5.32	2.46	1.98	1.61	4.10	5.78	8.33	4.93	3.26
Saccade amplitudes (° vf)	0.03	0.08	0.12	0.21	0.10	0.08	0.01	0.05	0.10	0.19	0.09	0.08
Coefficient K	0.00	0.01	0.02	0.04	0.02	0.02	0.01	0.02	0.03	0.05	0.03	0.02
Time spent in ambient mode (ms)	8.76	25.36	46.42	60.49	35.59	20.14	7.22	22.81	39.77	65.25	34.53	25.54
Number of modes switches	0.09	0.19	0.43	0.71	0.45	0.27	0.14	0.29	0.38	0.75	0.41	0.27
Fixation dispersions on X axis (pixels)	3.15	4.57	5.77	8.64	5.66	2.50	0.61	1.36	2.66	4.79	2.54	1.76
Fixation dispersions on Y axis (pixels)	0.92	1.77	2.62	3.44	2.28	1.04	0.38	1.46	3.57	5.49	3.65	2.17

For each metric and each effect size category, the values of the associated regression coefficients *β* are indicated. The means and standard deviations are for all the regression coefficients *β* computed for each metric.

#### Openness to experience

For the Openness to Experience trait for images, the signature for fixation durations was not robust over time, being only present in the final second, *β* = − 18.64, *p* < 0.005. The signature related to the number of mode switches remained constant, appearing in 8 out of 10 s, *β* = 1.16 ± 0.08, *p* < 0.001. No signature of this Openness to Experience trait was observed when dissecting its effects over time in the target-finding condition and on the web pages. As shown in Fig. 3 (part b), the effect of the Openness to Experience trait is not robust across task and stimuli. There is therefore no GSP for the trait of Openness to Experience.

#### Extraversion

For analyses on images, a significant effect was observed at the 8th second and 4th second for the Extraversion trait on the time spent in ambient mode for the free-viewing and the target-finding tasks *β* = 121.15 ± 14.91, *p* < 0.001 The dispersions along the X axis also showed a positive significant effect of the Extraversion personality trait, but only for the target-finding task, *β* = 13.3 ± 1.26, *p* < 0.001. No GSPs associated with the Extraversion trait were observed on the web pages. In addition, as shown in Fig. 3 (part b), the effect of the Extraversion trait is not robust across task and stimuli. Hence, there is no GSP associated with the trait of Extraversion.

#### Conscientiousness

For Conscientiousness across both tasks on images, constant and robust fixation durations and saccade amplitudes over time were found, with *β* = 6.15 ± 5.16, *p* < 0.001; and *β* = 0.13 ± 0.05, *p* < 0.001, respectively. The constancy and robustness of the other metrics varied depending on the task. Specifically, the K coefficient was more robust during free-viewing, evidenced by *β* = − 0.02 ± 0.04, *p* < 0.001, compared to target-finding with *β* = − 0.01 ± 0.04, *p* < 0.001. However, it remained inconstant across both tasks. The metrics for time spent in ambient mode, number of mode switches, dispersion along the X-axis and Y-axis were far more robust and constant during target-finding, with *β* = − 34.42 ± 28.48, *p* < 0.001; *β* = − 0.60 ± 0.15, *p* < 0.001; *β* = − 2.42 ± 2.22, *p* < 0.001; and *β* = − 3.39 ± 0.75, *p* < 0.001, respectively, than during free-viewing, where the results were *β* =14.43 ± 0.08, *p* < 0.001; *β* = 0.30 ± 0.08, *p* < 0.001; *β* = − 0.10 ± 1.31, *p* < 0.001, and *β* = 0.13 ± 1.42, *p* < 0.001, respectively. For the analyses on web pages, fixation durations, *β* = 3.15 ± 4.01, *p* < 0.001; saccade amplitudes, *β* = 0.17 ± 0.08, *p* < 0.001; number of mode switches, *β* = 0.31 ± 0.10, *p* < 0.001; and the dispersion of fixations on the Y-axis, *β* = 4.09 ± 1.01, *p* < 0.001, were constant and robust across both tasks and over time. Conversely, the K coefficient was more robust and constant during free-viewing, *β* = − 0.02 ± 0.02, *p* < 0.001, than during target-finding, *β* = 0.01 ± 0.04, *p* < 0.001. Regarding the time spent in ambient mode, higher constancy and robustness were observed during target-finding, *β* = 58.58 ± 15.46, *p* < 0.001, compared to free-viewing, *β* = − 12.95 ± 25.12, *p* < 0.001. Furthermore, as shown in Fig. 3 (part b), the effect of the Conscientiousness trait remained consistent across tasks and stimuli for fixation durations (task and stimulus robust), saccade amplitudes (task and stimulus robust), and the K coefficient (stimulus robust). Considering these results, there is a GSP for the trait of Conscientiousness for fixation durations and saccade amplitudes.

#### Agreeableness

For the analyses on images, fixation durations, dispersion of fixations on the vertical axis, and the K coefficient were more robust and constant over time during free-viewing, with *β* = − 14.31 ± 3.63, *p* < 0.001; *β* =− 3.47 ± 1.17, *p* < 0.001; and *β* =− 0.01 ± 0.04, *p* < 0.001, respectively, than during target-finding, with *β* =− 4.15 ± 2.50, *p* < 0.001; *β* = − 0.01 ± 1.78, *p* < 0.001; and *β* = − 0.001 ± 0.04, *p* < 0.001, respectively. For the time spent in ambient mode and the dispersion of fixations on the X-axis, the signatures remained robust and constant across both tasks, evidenced by *β* = 89.02 ± 52.45, *p* < 0.001 and *β* = 4.80 ± 1.16, *p* < 0.001, respectively. Regarding the number of mode switches, signatures were robust over time during free-viewing, *β* = 1.97 ±0.09, *p* < 0.001, as opposed to target-finding, *β* = 0.35 ± 0.0, *p* < 0.001. For the analyses on web pages, fixation durations, *β* = − 4.32 ± 3.69, *p* < 0.001; coefficient K, *β* = − 0.01 ± 0.04, *p* < 0.001; number of mode switches, *β* = 0.79 ± 0.08, *p* < 0.001; and dispersion of fixations along the horizontal axis, *β* = − 4.25 ± 1.19, *p* < 0.001, demonstrated the same level of robustness and constancy across both tasks. On the other hand, saccade amplitudes appeared to be more robust during free-viewing, *β* = 0.11 ± 0.04, *p* < 0.001, than during target-finding, *β* = − 0.01 ± 0.09, *p* < 0.001. The time spent in ambient mode and the dispersion of fixations along the vertical axis were less robust and constant in free-viewing, *β* = − 18.33 ± 0.00, *p* < 0.001 and *β* = 1.90 ± 0.62, *p* < 0.001, compared to target-finding, *β* = 57.46 ± 10.20, *p* < 0.001 and *β* = 5.06 ± 0.91, *p* < 0.001. As shown in Fig. 3 (part b), the effect of the Agreeableness trait remains consistent across tasks and stimuli for fixation durations and number of mode switches and task robust fixation dispersions on the X axis. Thus, there is a GSP for the trait Agreeableness for fixation durations, and the number of mode switches.

#### Neuroticism

Numerous signatures were observed for the trait of Neuroticism in images. Fixation durations exhibited greater constancy in signatures over time in target-finding, *β* = 7.13 ± 2.18, *p* < 0.001, than in free-viewing, *β* = − 2.01 ± 5.44, *p* < 0.001. For saccade amplitudes and time spent in ambient mode, robustness was higher over time during free-viewing, *β* = 0.14 ± 0.07, *p* < 0.001 and *β* = 44.13 ± 17.50, *p* < 0.001, respectively, than in target-finding, *β* = 0.09 ± 0.05, *p* < 0.001 and *β* = 27.07 ± 7.30, *p* < 0.001, respectively. Regarding the number of mode switches, greater constancy over time was observed in the target-finding task, *β* = − 0.42 ± 0.10, *p* < 0.001, than during free-viewing, *β* = − 0.24 ± 0.11, *p* < 0.001. Dispersion of fixations along the horizontal axis showed higher constancy over time in the target-finding task, *β* = 4.19 ± 1.75, *p* < 0.001, than in the free-viewing task, *β* = − 0.47 ± 1.09, *p* < 0.001. The dispersion of fixations along the vertical axis exhibited similar constancy and robustness over time through both tasks, *β* = − 1.14 ± 2.42, *p* < 0.001, although the directions were positive in the free-viewing task and negative in the target-finding task.

Lastly, the effect of the Neuroticism trait remained consistent across tasks and stimuli for saccade amplitudes but was only task robust for fixation durations and time spent in ambient mode as shown in Fig. 3 (part b). There is therefore a GSP for the trait Neuroticism for saccade amplitudes.

### Specific case of social content

Given the limited number of significant effects observed for the trait Extraversion, and considering its context-dependent nature, additional analyses were conducted to observe if further effects of the Extraversion trait emerged only for stimuli with social content. This characteristic of the stimuli being the most closely linked and evident for eliciting the Extraversion trait. Significant effects of Extraversion trait were found for stimuli containing a social content but restricted to images stimuli. On web pages, only one significant effect of Extraversion on the K Coefficient at the 3rd second was found in the target-finding task, *β* = − 0.14, *p* <0.05. On images, we observed significant effects of trait Extraversion in both free-viewing and target-finding tasks on fixation durations, *β* = 9.48±5.43, *p* <0.05 and *β* = − 6.42, *p* <0.05, saccade amplitudes, *β* = 0.24, *p* <0.05 and *β* = 0.07±0.03, *p* <0.05, the K coefficient, *β* = 0.008±0.06 and *β* = 0.02, *p* <=0.05, number of switches, *β* = 1.08±0.13 and *β* = 0.61±0.19), dispersion on the Y axis, *β* = 2.69±0.50, *p* <0.05 and *β* = 1.73±1.06, *p* <0.05 respectively. Significant effects of Extraversion were found on the time spent in ambient mode, *β* = 87.81±27.30) in the free-viewing task and on the Y-axis fixation dispersion in the target-finding task, *β* = 1.73±1.06, *p* <0.05 Despite these newly observed effects on social stimuli, few effects persist beyond 5 s (except for the dispersion of fixations along the X-axis for the target-finding task on images). As a result, no sustained GSP was truly identified.

## Discussion

The aim of the present study was to identify GSPs, which are, by nature, presumed to be constant and robust across various contexts and over time. By using linear regressions, in order to stay as close as possible to the initial theoretical framework of the Big Five model, analyses on scores were conducted trait by trait over several metrics in different contexts engaging bottom-up and top-down factors. Finally, by taking into account the dynamic nature of visual exploration and the two modes of visual processing, a GSP was found for three out of the five traits of personality. The following sections present the main results for each trait and discuss their implications.

The Openness to Experience trait imprinted a signature mainly on dynamic metrics during the free-viewing of images. This suggests that the more open-minded we are, the more time we spend in ambient processing and the more we alternate between the two exploration modes. The lack of observed signatures on web pages and target-finding on images hints that adding top-down task constraints or bottom-up stimulus constraints diminishes expression of the Openness trait. We hypothesize that the time constraints associated with the experiment may have prevented this trait from manifesting. Regarding the Extraversion trait, only limited significant effects were detected, specifically in the spatial information of fixation dispersions during image target-finding tasks. The greater horizontal spatial processing observed among extroverted individuals may hint at their broader visual environment processing due to their outward-facing nature. This broad exploration might be attributed to extroverted individuals’ inclination to be more engaged with their surroundings^43^. However, it is noteworthy that such signatures were absent during the free-viewing task of images or on web pages. This can be explained by the social orientation of the Extraversion trait. Given that the stimuli varied in their social content, with either the presence or absence of social agents such as faces or people, we ran further analyses focusing on social content. These analyses indeed showed more significant Extraversion trait signatures. This underscores the importance of the context for certain traits, as suggested before^24^. The singular presence of this trait during the target-finding task on images might have been influenced by our choice of stimuli, suggesting that to truly capture the essence of a trait such as Extraversion, the experimental setup needs to mirror its social inclinations. In addition, the traits Openness to Experience and Extraversion are not robust to the task or stimulus. These results indicate that these traits have little to no effect and that they are not stable (their direction of effect changes across tasks and stimuli).

Several explanations can be explored for the limited effects observed regarding the Openness to Experience and Extraversion traits, which usually exhibit notable effects on eye movements^34,45^. As for the limited effects of Openness to Experience, they might be attributed to the specific task and stimulus content given to participants. In a previous study^34^, participants were tasked with browsing web pages and retrieving text-based information, which involves a visual processing more focused on text. In contrast, our study involved tasks of free-viewing and target-finding, where the target consisted solely of objects, not text. Surprisingly, no signature was found for web pages probably due to the too limited time (only 15 s), even in the free-viewing condition. This could explain why only the free-viewing on images allowed this curiosity-associated trait to reveal itself. Concerning the weak effects of the Extraversion trait we found, the Al-Samarraie et al. study^34^ showed that individuals with higher Extraversion scores exhibited more distinct eye movements in an exploratory search task (involving interpretations beyond the information given or requested) than in a factual task (involving seeking specific information, such as the name of a place or product). This could explain the lack of significant effects of the Extraversion trait but only in the target-finding condition. Interestingly, GSP for the Extraversion trait emerged only for social stimuli showing that the expression of personality also depends on the context of visual exploration.

Turning to the Conscientiousness trait, the greatest robustness and temporal consistency were observed for the signatures during the target-finding task on images and web pages, with robust positive signatures across fixation durations and saccade amplitudes and negative signatures for all other metrics. This suggests that the more conscientious individuals are, the more they tend to exhibit deep visual processing, with a broader attentional scope and sequential (due to the negative effect on fixation dispersions) and stable visual exploration modes (due to the negative effect on the number of mode switches) in pursuit of optimal performance in the target-finding task. In free-viewing, fixation durations, saccade amplitudes, coefficient K and number of mode switches (across both stimuli) show positive signatures of the Conscientiousness trait. In this task, it can be inferred that the more conscientious the participants are, the deeper and the more dispersed their processing becomes, with a wide span of visual-spatial attention and more dynamic alternation between ambient and focal modes.

The robustness of the Conscientiousness trait demonstrates that both information processing indicators (fixation durations, with a positive effect), and visual-spatial attention deployment (saccade amplitudes, with a positive effect) are robust to task and stimulus variations (see Fig. 3). This personality trait expresses itself consistently regardless of whether there is a modification in a high-level factor (task) or a low-level factor (stimulus). The indicator of exploration mode used (coefficient K, with a positive effect) is robust to the stimulus but not to the task. A significant factor influencing the manifestation of the Conscientiousness trait is the task, which encourages constant visual behaviors. The importance of this trait in the experimental design is evident, especially since participants were required to accomplish tasks in an evaluative context, shedding light on its more frequent appearances. For the trait of Agreeableness, when executing the target-finding task on both images and web pages, positive signatures were found on dynamic visual exploration indicators (such as time spent in ambient mode and the number of mode switches), while negative signatures were observed on fixation durations. This suggests that individuals scoring higher on Agreeableness tend to engage less in deep visual processing, spending more time in ambient mode and switching between visual modes more frequently in their attempts to complete the target-finding task. In the free-viewing condition, consistent negative signatures were found on fixation durations (both images and web pages), coefficient K (only for images), fixation dispersions on the X-axis (for web pages) and the Y-axis (for images). Conversely, positive consistent signatures were found on saccade amplitudes (for web pages), dispersion on Y-axis (for web pages), and time spent in ambient mode, number of mode switches and dispersion on the X-axis (for images). To encapsulate the main findings, these results demonstrate that in free-viewing, more agreeable individuals exhibit superficial processing, with attention scattered over simple stimuli (for images), an increased alternation of exploration modes across both tasks and stimuli, and a task robustness link with fixation dispersions on the X-axis. The robustness of the trait Agreeableness demonstrates that both information processing indicators (fixation durations, with a negative effect) and gaze stability (number of mode switches with a positive effect) are robust to task and stimulus variations. The visual exploration mode (fixation dispersions on the X-axis) is robust to task variations but not to stimulus variations. The study’s specific context may have made participants more inclined to cooperate, following study directives more closely and genuinely engaging with the stimuli. They might have been more motivated to truly explore when searching for a target and to delve deeper when tasked to locate them. These two findings for Conscientiousness and Agreeableness traits support previous studies^29,34^, showing that Agreeableness and Conscientiousness can affect eye movement parameters. Lastly, for the Neuroticism score, we observed a slightly better robustness and temporal consistency in signatures during the target-finding tasks on both images and web pages. Positive signatures were evident on fixation durations and saccade amplitudes (for both stimuli) and positive signatures for fixation dispersions on the X-axis (for web pages) and negative signatures for number of mode switches and fixation dispersions on the Y-axis (for images). For the free-viewing task, we observed a positive signature of Neuroticism on saccade amplitudes and time spent in ambient mode (both stimuli). A positive signature was found on fixation dispersions on the X-axis (for web pages) and a negative signature was found on K coefficient (for images). More generally, there appear to be GSPs primarily for the depth in processing (fixation duration) as already observed^34^ and deployment of visual-spatial attention (saccade amplitudes), independently of the task or stimulus modality. Indeed, as showed previously^34^, we also observed differences in personality markers depending on the task, showing that personality is not expressed in the same way through eye movements depending on the participant’s task. Findings lend further credence to the observations made in previous study^46^, who showed that the nature of tasks results in different information-seeking modes. This observation was further confirmed by^34^, who observed that individuals with high scores in Agreeableness tend to process information with fewer fixations and longer durations to retrieve the needed information. Conversely, individuals high in Extraversion were found to require shorter durations for information retrieval, suggesting that Extraversion significantly influences the speed of performing online exploratory tasks. This hierarchy of effects, with Extraversion leading, followed by Agreeableness and then Conscientiousness, in their results conducted them to interpreting a nuanced interplay between personality traits and eye movement parameters in information-seeking behaviors. In addition, results also showed a slight tendency to be more GSP task robust on web pages (4 GSPs for Conscientiousness, 4 for Agreeableness, and 2 for Neuroticism) than on images (2 GSPs for Conscientiousness, 4 for Agreeableness, 1 for Neuroticism). These results are in accordance with the findings of Berkovsky et al.^33^, which demonstrated that personality is more expressive in dynamic stimuli (videos) than in static stimuli (images). Supporting the assumptions of Sutin et al.^24^, and the claims of Wilt et al.^47^, showing that the emergence of personality effect depends on the context of visual exploration. More broadly, here the occurrence of GSP can be interpreted through the lens of cognitive functions, drawing upon the research conducted by Sutin et al.^24^, which delineates distinct markers of Conscientiousness, Agreeableness, and Neuroticism within cognitive processes. They showed that individuals with high Neuroticism showed lower performance across all cognitive tasks, suggesting Neuroticism’s impact on the five domains (Memory, Speed-Attention-Executive, Visuospatial Ability, Fluency, and Numeric Reasoning) evaluated. Here, three of them (Memory, Speed-Attention-Executive, and Visuospatial Ability) are likely involved. This significant association with Neuroticism and cognitive processes confirms the potential to find signatures of this trait in eye movements, as they are linked to information processing^48^. Similarly, Conscientiousness trait showed correlations with high performance tested in all subdomains, as did Agreeableness trait (except for numeric reasoning), which also explains why most of the signatures were found for these traits.

The results show that conducting analyses over time allows for the observation of more personality signatures. Indeed, some signatures are not constant and robust over time, which prevents them from emerging when we conduct analyses by chunking the data by trial. The discrepancy between the results of analyses by trial, which show minimal GSP, and those of temporal analyses, underscores the significance of an appropriate chunking method. As demonstrated in our study and others^4,5^, time significantly impacts the evolution of ocular metrics, which reflect different kinds of information processing. Using a 1 s chunking method appears to yield more constant effects within the chunking window, revealing significant relationships. Previous studies have emphasized the importance of chunking methods in the study of links between continuous behavioral output and cognitive processes^49,50^. For instance,^49^ studied the effect of arousal and valence on mouse interactions and found it beneficial to chunk data every 500 ms “to provide the most appropriate level of detail from the data”^49^. A potential explanation for this phenomenon might be the visual processing modes used. It is plausible that relationships between personality and eye movements exist in one direction within a particular processing mode (ambient/focal) and might disappear or reverse in another (focal/ambient). Thus, by chunking data over time, we analyze data from the same mode, in which relationships are constant between behavioral output and cognitive processes. Interestingly, top-down and bottom-up factors seem sometimes to reduce the markers of personality or even reverse their effects. Specifically, the traits Openness to Experience and Extraversion are the ones that show the fewest personality signatures. Conversely, the traits Conscientiousness, Agreeableness and Neuroticism are the ones that contain the most signatures across the task, stimulus, and time. Among the observed GSPs, findings suggest that the more conscientious we are, the deeper our processing becomes, coupled with a dispersed visuo-spatial attention.

Echoing our remarks on the Extraversion and Openness traits, the Conscientiousness trait seems more pertinent since we asked participants to accomplish tasks with objectives in an evaluative context. This may partly explain why we see more signatures for this trait. There are certain limitations to the study that should be pointed out. We demonstrated that the traits of Conscientiousness, Agreeableness, and Neuroticism are the personality traits that show the most gaze-based signatures, while the traits Openness to Experience and Extraversion are those that show the fewest.

However, it should be remembered that the present study was conducted in the laboratory, with limited time to perform the two visual tasks on both image and web page stimuli. The experimental context may lead to revealing more personality signatures for these three traits. Indeed, the trait of Conscientiousness is probably relevant to visual exploration tasks. The Agreeableness trait, linked to an individual’s conciliatory ability, might also have been more expressed due to task demands, perhaps revealing the participants’ willingness to cooperate. Finally, the Neuroticism trait, associated with anxiety and depression, indicates a level of emotional management. Given that the laboratory research context is potentially stressful for participants, this setting might have accentuated the expression of this trait. On the contrary, the trait of Extraversion, which pertains to Openness to Experience and ease of interaction in a social context, is not necessarily relevant in a laboratory research environment. Similarly, Openness to Experience, linked to open-mindedness and curiosity, might not be as relevant in performing a time constrained task in the laboratory. We believe that this research context could potentially have reduced the expression of the latter two traits. Efforts should be made to minimize the researcher’s presence, potential stress factors and time constraints to ensure a better capture of the gaze-based signature of personality.

To conclude, we have introduced the concept of the gaze-based signature of personality (GSP) by proposing a new method to unveil these GSPs through varied exploration contexts, analyzing eye movements using second-by-second data chunking. The analyses have provided encouraging results concerning these distinct personality signatures in eye movements, highlighting the ways in which individual nuances can manifest in how we visually explore our environment. Further studies are needed to better characterize the conditions necessary for a signature of personality to emerge. However, this study demonstrates that it is possible to capture an gaze-based signature of personality from eye movement recordings in just a few seconds, encompassing around thirty eye movements, using simple tasks and stimuli reminiscent of everyday life. These findings pave the way for broad practical implications. If personality traits can be detected through eye movements, it is conceivable that other high-level factors, such as neuro-cognitive disorders or specificity, might also exhibit specific gaze-based signatures. Potential applications in neuropsychology, where early and implicit detection of disorders could be guided by eye movement analyses, can be envisaged. This study confirms the importance of emulating research aimed at highlighting context-robust markers. This could revolutionize diagnostic approaches and mark a significant shift in the clinical field.

## Methods

### Participants

Here, we studied the eye-movements of 91 participants (77 females, 12 males and 2 of other gender) aged between 18 yo and 38 yo (M = 20.05, SD = ±3.83 yo). Participants reported normal or corrected to normal vision and were naive about the purpose of this study. We verified if they were right-handed or able to use a computer mouse with the right hand. All participants were undergraduate students from the psychology institute at Université Paris Cité. Participants were compensated by course credit. All procedures and the experimental protocol conducted in this study, since it involves human participants, were carried out in accordance with the ethical guidelines outlined in the Declaration of Helsinki (1964) and approved by the local Ethics Committee of Université Paris Cité (No. CER-PD:2022-103). All participants provided written informed consent.

### Apparatus

Eye movements were recorded using an Eye-Link 1000 Plus (SR Research Ltd., Canada) at a 1 KHz sampling rate with 0.05° of spatial precision. We recorded the right eye of the participants with a 35 mm monocular lens. Stimuli were displayed on a 24.5 inch LCD computer screen with a 1920 × 1080 pixels resolution and a 240 Hz refresh rate. The experiment was run using WebLink (SR Research Ltd., Canada) and Mozilla Firefox 118.0.2. For the measurement of personality traits, we used the French Big Five Inventory (BFI-Fr) questionnaire with 45 items^43^, which are distributed respectively as 10, 9, 9, 10, and 8 items for Openness to Experience, Conscientiousness, Extraversion, Agreeableness, and Neuroticism traits.

### Stimuli

**Figure 4:**
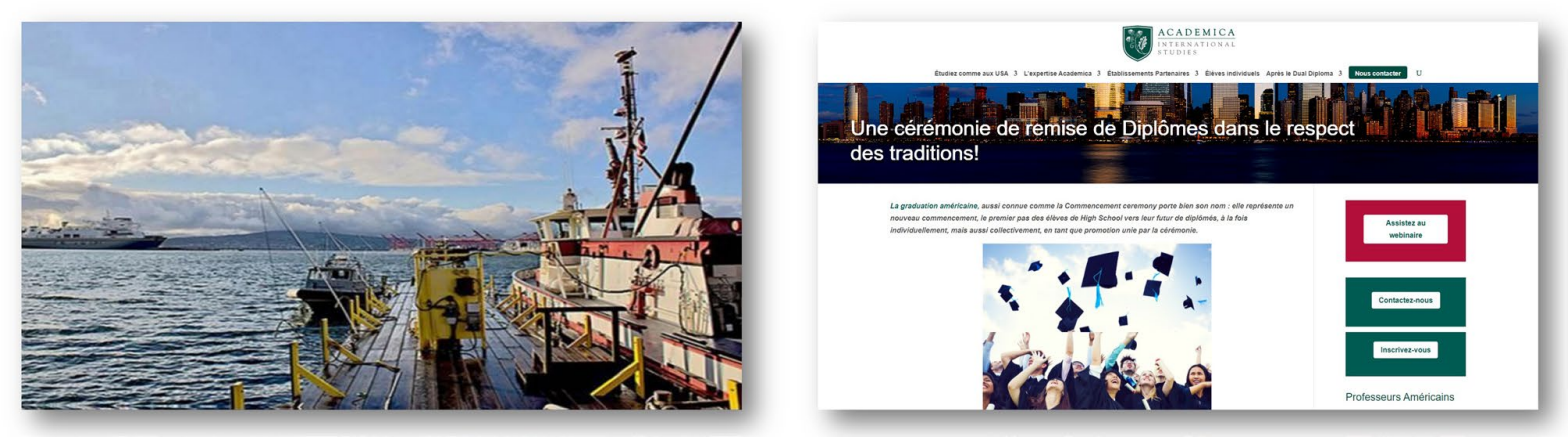
Examples of stimuli presented to participants. On the left is an example of an image from the COMPASS database^51^ and on the right is the first screen of a web page presented to participants (https://dualdiploma.org).

In this experiment, 24 images from the Complexe Affective Scene Set (COMPASS) database^51^ and 24 web pages from 24 different websites were randomly presented to the participants (see examples in Fig. 4) and already loaded locally. The COMPASS database is a set of images whose valence (negative, neutral and positive) and the presence of faces or people has been tested and validated. In this study, we presented only positive or neutral images. In each of these image categories, faces or people were present in some scenes and not in others. The image sizes were artificially increased to fit in a 1920 x 1080 pixels area. The web pages had a width of 1920 pixels and their total height was between 2838 pixels and 28100 pixels (M = 8668.29 px, SD = ± 6924.22 px). Participants were allowed to freely move the mouse, scroll, or click. However, hyperlinks and content animations were deactivated to prevent participants from leaving the web page displayed. The web pages and their topics were arbitrarily chosen, and included website source, front pages, textual pages, and articles. We ensured that each web page selected followed several criteria to minimize biases. The first criterion was that the web pages were in French to ensure that they were in a language familiar to the participants. The second criterion concerned the web pages’ news content. Since this study was run over several months, we excluded web pages that had content referring to current events or content related to a season, date, holiday, celebration, etc. As the third criterion, we checked that the web pages did not have any external advertising. Specifically for web pages, we also made sure that a balance was maintained for images, texts, general layout, and total length of the web page to have stimuli with different content types and organizations. Finally, as described in the following section, the targets to be found in the target-finding tasks already present within the original web page. Like images, web pages were split into 4 categories: neutral emotional content and no social content, neutral emotional content and social content, positive emotional content and no social content and positive emotional content and social content. There was an equal number of positive and neutral stimuli across the stimulus type (12 neutral images, 12 positive images, 12 neutral web pages, 12 positive web pages). Positive stimuli induced significantly more positive declared felt valence gathered from the Self Assessment Manikin than neutral ones for both images (neutral = 6.2 ± 1.1; positive = 6.32 ± 1; *β* = 0.12, *p* <0.05 and web pages (neutral = 6.16 ± 1.1; positive= 6.43 ± 1; *β* = 0.27, *p* < 0.05. Participants were randomly assigned to start with an image block or a web page block; then, each participant executed one task per stimuli during 15 s. This duration was chosen to avoid boredom due to lengthy exploration of an image.

### Tasks

Participants performed a total of 24 free-viewing tasks and 24 target-finding tasks over all images and web pages. Tasks and stimuli associations were generated with a random uniform distribution of probabilities. Each stimulus had an equal chance of being presented either as a free-viewing task or as a target-finding task. The participants did not know how many targets there were Table 2 but we informed them that there was at least one target per stimulus. As previously defined, the targets were icons or images present on the original stimulus. For both images and web pages, the chosen targets were initially part of the stimulus. Targets were selected by two judges (members of the laboratory) in order to have different levels of target detection difficulty, particularly due to their size, visual salience, number of targets and spatial positions within the stimulus. For a given stimulus, the targets and their number were always the same to find. The targets were equally distributed between the top, middle, and bottom of the stimulus and, specifically for web page, could be found on the sides, or in the content, header, or footer. The average number of targets were 4.72 ± 2.68 (from 1 to 14 targets to found) for images and 8.39 ± 2.42 (from 1 to 25 targets to found) for web pages. Participants did not receive any feedback upon clicking (or not clicking) on a target. This absence of feedback was deliberate, aiming to prevent the introduction of attentional biases, such as the distraction caused by unexpected popups^52^, during or after the clicking action.

**Table 2:** Descriptive statistics of number of targets to find for each stimulus type.

Stimulus type	Mean	Std	Min	25	50	75	Max
Web page	8.39	5.55	1	4	7	12	25
Image	4.72	2.29	1	2	3.5	7	14

### Experimental design

After obtaining participants’ informed consent, they were asked to fill out a socio-demographic questionnaire. Their initial emotional state was assessed using the Self Assessment Manikin (SAM) scale^53^. A 9-point calibration of the eye tracker was then introduced. Participants first undertook the free-viewing task, followed by the target-finding task. The task sequence was randomized among participants, and the presentation order of stimuli, whether static (image) or dynamic (web page), was counterbalanced. After viewing each stimulus for a total duration of 15 s, participants were shown the SAM scale to evaluate the emotional impact of the stimulus. After viewing all the stimuli, participants answered the French version of the BFI-45^43^.

### Data cleaning and analysis

We used a saccade detection threshold of 30° per second. If an eye movement speed exceeds this threshold, the eye-tracker labels a saccade; otherwise, it labels a fixation. For the data preprocessing, fixations less than 100 milliseconds long and them that precede or follow blinks were discarded^54^. The positions of fixations were corrected due to positional errors that can occur with fixations made at the screen edges. Fixations occurring outside the screen border (distance >3° of visual angle) were removed. Meanwhile, those made just outside the screen border, within a distance of less than 3°, were adjusted to the edge of the screen. Consequently, 98% of the initial fixations (140 178 fixations) of the 91 participants were retained. Data recorded during the first 10 s of visual exploration of each trial were selected to analyze. This duration was determined based on post-hoc analyses of the times at which the last target was clicked during the trial. The first 10-s duration proved to be adequate for obtaining data that reflected an always engaged target-finding behavior. This resulted in a satisfactory but not optimal number of targets identified: 85% of targets (mean of 4) were found in images and 34% of targets (mean of 2.3) were found on web pages within the first 10-s window. Second, we performed a data cleaning process to remove outliers from the total of 137,375 remaining fixations. To accomplish this, we employed the inter-quartile range (IQR) method^55^, opting to exclude data that were distant from the median by more than 3 times the IQR. This threshold is larger than the commonly used value of 1.5, but was chosen here because the data were collected in a particularly controlled environment and thus had a low likelihood of being inconstant. Therefore, we chose a more lenient approach, aiming to remove as few data points as possible. Moreover, upon evaluating various threshold values, we found it more appropriate to use an IQR threshold of 3 instead of 1.5, as the latter seemed overly restrictive. After this data cleaning phase, 3.56% of data were removed.

**Figure 5:**
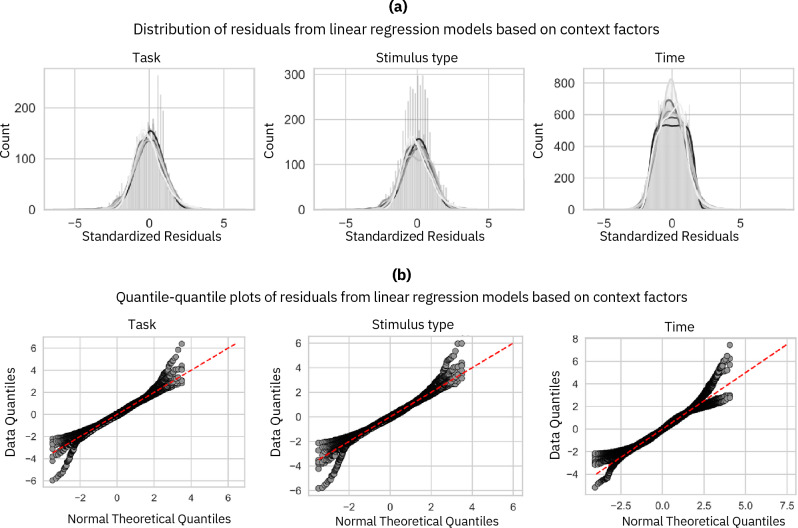
Residuals from linear regression computed for each context factor. The distribution of each eye movement indicator is related to the context factors. The histograms in section (**a**) above serve to visually inspect whether the residuals follow a bell-shaped curve. In contrast, the qq-plots in section (**b**) below visually compare the residuals to a theoretical normal distribution, with a straight line along the diagonal indicating a match with normal distribution characteristics.

**Figure 6:**
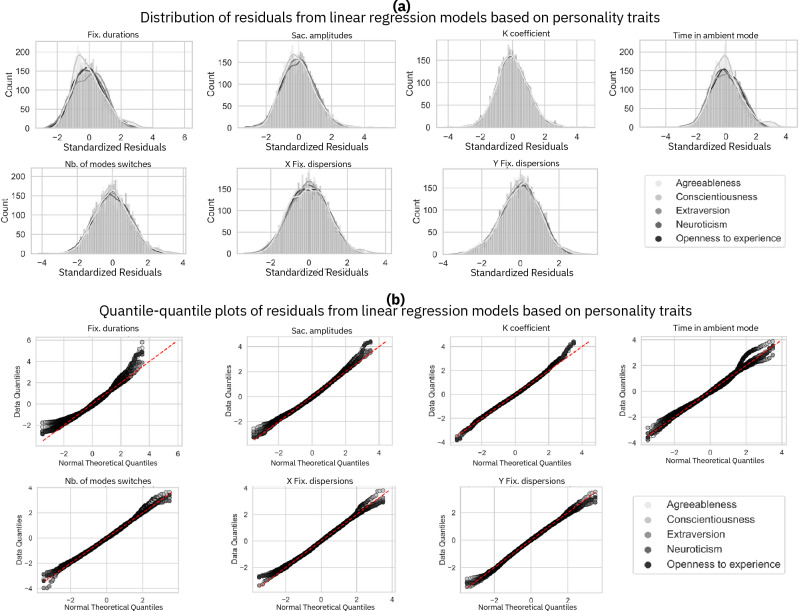
Histograms and quantile–quantile plots of residuals from linear regression computed for each context factor. Each graph represents residuals computed for one eye movement indicator for all the five personality traits. The histograms in the upper section (**a**) provide a visual assessment of whether the residuals form a bell-shaped distribution. The qq-plots in the lower section (**b**) depict residuals against a theoretical normal distribution, which should align along the diagonal line if they conform to a normal distribution.

After removing outliers, we ensured that the residuals from the linear regression models were normally distributed^56^. To achieve this, we conducted a Shapiro–Wilk test, which tests the null hypothesis that the observed data are normally distributed. This test is among the most recommended (Patricio, 2016)^57^. To compute residuals, we constructed regression models using eye movement indicators as the predicted variable and context and personality trait factors as predictors. A separate model for each predictor-predicted variable pair was built. Subsequently, a Shapiro–Wilk test was applied to the residuals of each predictive model with *α *≤ 0*.*05 to control for potential Type I error. This *α* < 0*.*05 threshold was selected for all data analyses conducted in this study. A *α* < 0*.*005 threshold was set for analyses involving time effects with the Bonferroni correction. As shown in Table 3, the distribution of residuals differed significantly for nearly all the computed residuals.Only the residuals from two models using Conscientiousness traits as the predictor (time spent in ambient mode and number of mode switches as the predicted variables) and one model using the Extraversion trait as the predictor (fixation dispersions on the X axis as the predicted variable) did not significantly deviate from a normal distribution at *α *≤ 0*.*05. However, these tests have limitations and may lead to incorrectly rejecting the null hypothesis (type II error)^57^. Therefore, we also opted to visually inspect the distribution of residuals^58^. This examination was conducted using quantile-quantile plots (qq-plots) and histograms of residuals^59^. In Fig. 5, we observe that only for the time factor, extreme values of residuals deviate from the theoretical value. For all other linear regression models, we can see from the histograms that the residuals exhibit a bell-shaped distribution and conform to a normal distribution according to the qq-plots. Considering the data, and given that linear regression models are considered robust to violations of the normality assumption^60–65^ small deviations of the residuals at extreme values should not be of concern. The observations in Fig. 6 indicate that residuals follow a normal distribution for each eye movements metrics and each personality trait. However, for fixation durations, residuals do not conform to a theoretical normal distribution at extreme values. Based on these results, we conclude that the residuals of the linear model used to examine the effect of personality traits on each eye movements indicators are normally distributed. As for context factors, considering the data and the robustness of the linear regression models employed to violations of the normality assumption^64,65^, small deviations of the residuals at extreme values should not raise concerns.

**Table 3:** *P*-values from Shapiro–Wilk tests conducted on the residuals of each linear regression model with the relative predictor and predicted variables.

	Fix. durations	Sac. amplitudes	K Coefficient	Time in ambient mode	Nb. of modes switches	X Fix. dispersions	Y Fix. dispersions
Task	5.92e − 36	4.95e − 21	6.43e − 10	2.85e − 31	3.10e − 09	2.93e − 16	3.50e − 13
Stimulus type	1.66e − 35	1.15e − 20	5.97e − 10	7.69e − 31	1.09e − 06	3.61e − 08	6.68e − 05
Time	0.00e + 00	0.00e + 00	0.00e + 00	0.00e + 00	7.57e − 43	8.20e − 41	6.37e − 34
Openness to experience	3.99e − 41	2.72e − 21	3.85e − 09	3.42e − 35	7.21e − 17	2.04e − 13	1.43e − 04
Conscientiousness	1.01e − 20	1.18e − 10	4.31e − 09	7.85e − 02	5.14e − 01	2.02e − 06	1.23e − 08
Extraversion	1.70e − 09	2.07e − 06	1.98e − 08	1.33e − 09	8.45e − 03	5.05e − 02	9.89e − 08
Agreeableness	3.97e − 26	1.61e − 18	7.03e − 09	1.09e − 08	3.60e − 02	6.01e − 10	1.09e − 07
Neuroticim	7.88e − 11	2.27e − 05	1.91e − 09	9.37e − 12	1.45e − 05	1.90e − 05	1.54e − 08

## Data Availability

The dataset used and analyzed in this study is available from the corresponding author upon request without undue reservation.
